# Design specifications for biomedical virtual twins in engineered adoptive cellular immunotherapies

**DOI:** 10.1038/s41746-025-01809-6

**Published:** 2025-08-01

**Authors:** Ulrike Weirauch, Markus Kreuz, Colin Birkenbihl, Miriam Alb, Maria Quaranta, Laurence Calzone, Sophia Orozco-Ruiz, Stefanie Binder, Luise Fischer, Solène Clavreul, Morine Maguri, Maximilian Ferle, Michael Rade, Guillaume Azarias, Jay R. Hydren, Jakub Jamarik, Daniel Schwarz, Zsolt Sebestyen, Jurgen Kuball, Georg Popp, Chloé Antoine, Manon Knockaert, Clara T. Schoeder, David Fandrei, Carmen Sanges, Vaclovas Radvilas, Nico Gagelmann, Markus Rückert, Olaf Penack, Stephan Fricke, Andreas Schmidt, Carol Ward, Carl Steinbeisser, Jean-Marc Van Gyseghem, Anna Niarakis, Laurent Garderet, Michael Hudecek, Thomas Neumuth, Uwe Platzbecker, Ulrike Köhl, Regina Demlova, Andreas Kremer, Stefan Franke, Holger Fröhlich, Maximilian Merz, Kristin Reiche, On behalf of the CERTAINTY Consortium, Ulrike Weirauch, Markus Kreuz, Colin Birkenbihl, Miriam Alb, Maria Quaranta, Laurence Calzone, Sophia Orozco-Ruiz, Stefanie Binder, Luise Fischer, Solène Clavreul, Morine Maguri, Michael Rade, Guillaume Azarias, Jay R. Hydren, Jakub Jamarik, Daniel Schwarz, Zsolt Sebestyen, Jurgen Kuball, Georg Popp, Chloé Antoine, Manon Knockaert, Clara T. Schoeder, David Fandrei, Carmen Sanges, Vaclovas Radvilas, Nico Gagelmann, Markus Rückert, Olaf Penack, Stephan Fricke, Andreas Schmidt, Carol Ward, Carl Steinbeisser, Anna Niarakis, Laurent Garderet, Michael Hudecek, Thomas Neumuth, Uwe Platzbecker, Regina Demlova, Andreas Kremer, Stefan Franke, Holger Fröhlich, Maximilian Merz, Kristin Reiche, Maximilian Ferle, Jean-Marc Van Gyseghem, Ulrike Köhl

**Affiliations:** 1 Fraunhofer Institute for Cell Therapy and Immunology IZI, Leipzig, Germany; 2 Fraunhofer Institute for Algorithms and Scientific Computing SCAI, Sankt Augustin, Germany; 3 Department of Internal Medicine II, Chair of Cellular Immunotherapy, University Hospital Würzburg, Würzburg, Germany; 4 Information Technology for Translational Medicine (ITTM) S.A., Esch-sur-Alzette, Luxembourg; 5 Institut Curie, INSERM, U 1331, Mines Paris Tech, PSL Research University, Paris, France; 6 INSERM, U900, Paris, France; 7 Institute for Clinical Immunology, University Hospital of Leipzig, Leipzig, Germany; 8 Department of Hematology, Hemostaseology and Cellular Therapy, University Hospital of Leipzig, Leipzig, Germany; 9 Myeloma Patients Europe AISBL, Brussels, Belgium; 10 Innovation Center Computer Assisted Surgery, Universität Leipzig, Leipzig, Germany; 11 Center for Scalable Data Analytics and Artificial Intelligence (ScaDS.AI), Dresden/Leipzig, Germany; 12 TriNetX Oncology GmbH, Freiburg, Germany; 13 HealthTree Foundation Inc., Lehi, UT USA; 14 CREATIC, Faculty of Medicine, Masaryk University, Brno, Czechia; 15 Center for Translational Immunology and Department of Hematology, University Medical Center Utrecht, Utrecht University, Utrecht, The Netherlands; 16 Cellular Therapy and Immunobiology Working Party (CTIWP) of the European Society for Blood and Marrow Transplantation (EBMT), Leiden, The Netherlands; 17 Research Center Information, Law and Society, University of Namur, Namur, Belgium; 18 Institute for Drug Discovery, Faculty of Medicine, Leipzig University, Leipzig, Germany; 19 European Society for Blood and Marrow Transplantation (EBMT), Leiden, The Netherlands; 20 Department of Stem Cell Transplantation, University Medical Center Hamburg-Eppendorf, Hamburg, Germany; 21 Chronic Malignancy Working Party, Multiple Myeloma Sub-committee Chair CAR-T, of the European Society for Blood and Marrow Transplantation (EBMT), Leiden, The Netherlands; 22 Department of Hematology, Oncology and Tumorimmunology, Charité—Universitätsmedizin Berlin, Freie Universität Berlin and Humboldt-Universität zu Berlin, Berlin, Germany; 23 Medicine Campus MEDiC of the Dresden University of Technology at Klinikum Chemnitz gGmbH, Chemnitz, Germany; 24 Singleron Biotechnologies GmbH, Cologne, Germany; 25 F. Hoffmann-LaRoche AG, Basel, Switzerland; 26 Collaborate Project Management, Munich, Germany; 27 Molecular, Cellular and Developmental Biology Unit (MCD), Centre de Biologie Integrative (CBI), University of Toulouse, UPS, CNRS, Toulouse, France; 28 Lifeware Group, Inria, Paris, France; 29 Hematology and Cellular Therapy Department, Hopital Pitié Salpêtrière APHP, Sorbonne Université, Paris, France; 30 Fraunhofer Institute for Cell Therapy and Immunology IZI, Cellular Immunotherapy Branch Site Würzburg, Würzburg, Germany; 31 Bonn-Aachen International Center for IT (b-it), University of Bonn, Bonn, Germany; 32 Myeloma Service, Memorial Sloan Kettering Cancer Center, New York, USA

**Keywords:** Computational models, Data integration, Machine learning, Software, Haematological diseases, Biomarkers, Health care, Medical research

## Abstract

In (immune)oncology, virtual twins (VTs) offer patient-individual decision support. Nevertheless, current VTs do not incorporate the unique properties of engineered adoptive cellular immunotherapies (eACIs). Here, we outline the minimal design specifications for VTs for engineered ACIs (eACI-VTs) to model the complex interplay between cell product and patient physiology. We motivate utilizing VTs in eACIs to provide decision support and reflect on how eACI-VTs can support the widespread use of eACIs.

## Introduction

Adoptive cellular immunotherapy (ACI) is a novel therapy with the potential to revolutionize the treatment of cancer and other diseases^1^. Prominent examples include engineered ACIs (eACIs), such as chimeric antigen receptor (CAR) T cells and T cell receptor (TCR) engineered T cells, as well as non-eACI-like tumor infiltrating lymphocytes^2^, with CAR T cells being the most prevalent eACI in current clinical practice. CAR T cell therapy equips a patient’s own (autologous) or a healthy donor’s (allogenic) T cells with CARs, enabling them to recognize a defined target antigen on the surface of tumor cells^3^. Upon encountering tumor cells, CAR T cells become activated and are thereby enabled to kill the target cells. Six autologous CAR-based therapies are approved by the European Medicines Agency (EMA), and seven by the United States Food and Drug Administration (FDA), for hematological malignancies. With CAR T cells, an unprecedented proportion of patients experience long-lasting remission or even cure, with manageable adverse events^4,5^. However, not all patients respond to treatment^5^. Predicting which patients will benefit from eACI is crucial due to outcome uncertainty, high therapy costs, and limited manufacturing capacities, because the cell product is manufactured individually through a complex, multi-step process.

In healthcare, digital twins (DTs) and virtual twins (VTs) are computer-based models that digitally represent interacting biological systems across multiple scales. These models support monitoring disease prevention, diagnosis, treatment decision-making, and follow-up care, while also assisting both clinical and nonclinical research, thus accelerating the development of new medicines and medical devices. Although DTs/VTs are still in their early stages of development, their potential to transform precision medicine through individualized care is increasingly recognized^6–9^.

Definitions of the term DT vary in the literature and often lack a clear distinction from VTs. Viceconti et al. define a DT in healthcare as an application-specific virtual representation of a single organ of an individual patient, intended to guide patient-specific decisions and requiring integration with the patient’s personal data at least once during its life cycle^10^. Specifically, a digital representation of a real-world object qualifies as a DT if it includes: (i) a computational model of the object, (ii) a dataset describing changes in the object, and (iii) methods for continuously updating the computational model with data derived from its real-world counterpart^11^. Importantly, a DT is expected to evolve in parallel with its real-world counterpart. In healthcare, this real-world object may represent a patient, a clinical study participant (e.g., from a control arm), or any biological system, such as individual cells or organs. These definitions for DTs explicitly exclude population-based models because they lack continuous updates based on patient-specific data^10,11^. Expanding upon this, we define a VT in healthcare as an application-specific in silico system covering at least two single-organ DTs from the same patient to simulate multi-organ biomedical interplay. As such, VTs offer the complexity of different biological scales, dynamic adaptability, and different organs to guide treatment decisions. This makes VTs particularly well-suited for use in patients eligible for eACIs. Unlike conventional drugs, eACIs require modeling the medicinal product not only as a dynamic biological system of living cells but also in terms of its interplay with multiple organs of the patient. To motivate the development of VTs for patients eligible for eACIs (eACI-VTs), we propose minimum design specifications for such models. We use autologous CAR T cell therapy as a representative example, given its status as the most widely approved eACI. The main principle, however, readily extends to all classes of eACIs, such as allogenic CAR T cells, CAR NK cells, CAR macrophages, and TCR-engineered T cells, and can also be adapted to accommodate the distinct features of non-eACIs.

## Engineered adoptive cellular immunotherapies

### Current limitations for a broader application of engineered adoptive cellular immunotherapies

In 2017, Tisagenlecleucel (Kymriah®) was approved by the FDA as a first-in-class therapy, and in 2018 by the EMA, as a third-line treatment for acute lymphoblastic leukemia (ALL), follicular lymphoma, and diffuse large B-cell lymphoma (DLBCL). Since then, five additional products have entered the market in the European Union (EU) and six in the United States. These therapies target either Cluster of Differentiation 19 (CD19) in BCL and B-cell precursor ALL, or B-cell maturation antigen (BCMA) in multiple myeloma (MM).

Numerous studies aim to improve CAR T cell therapies (Box 1). Due to the unprecedentedly high therapy response rates in heavily pre-treated patients with hematological malignancies, efforts are underway to expand this therapy to earlier lines of therapy and additional indications, including solid cancers and beyond, particularly autoimmune and infectious diseases^12^. These advances coincide with a substantial increase in the number of treatment-eligible patients. Nevertheless, three main challenges already limit the availability of CAR immunotherapies: (i) the high cost of this treatment^13^, (ii) limited manufacturing capacity^3^, and (iii) the need for optimal patient stratification considering efficacy and safety^14,15^. It remains uncertain how significantly treatment costs will actually decrease with manufacturing automation, point-of-care production, or off-the-shelf allogenic cell products^3^, especially given the complex reimbursement landscape. Nevertheless, overcoming manufacturing limitations and standardizing production, along with improving treatment success, will critically depend on the development of individualized decision-support tools^16–18^. Specifically, patient-specific predictive models can provide objective criteria for the optimal timing of eACI therapy, helping to address critical manufacturing constraints through the timely allocation of manufacturing resources.

Box 1—Advances in engineered adoptive cellular immunotherapies CAR T cell therapy has shown unprecedented effects in hematological malignancies with overall manageable side effects. Nonetheless, cases of limited efficacy, severe toxicities, timely relapse, and therapy resistance persist, which require further product development. Sparked by their overall success, CAR-based therapies are also evaluated for other indications. Clinical studies treating solid cancer^103,104^, autoimmune disease^105,106^, and infectious disease^107,108^ show encouraging results. Here, similar but also indication-specific challenges are faced, necessitating indication-individual adjustments of eACIs. Major strategies to advance CAR-based therapies include:

**Improving CAR-design**: The CAR-design can be modularly adapted by adding or removing domains to improve the signaling after CAR activation for the anticipated functionality^109^. Furthermore, adjusting the affinity of the CAR to its antigen can increase specificity and safety^110^. Universal CAR platforms allow for a swift adaptation of the CAR targeting domain to varying target antigens^111,112^.

**Adjustments on the immune cell level**: Current approved CAR-based therapies use autologous T cells. To increase the efficacy of these products, there are approaches to select only those CAR T cell populations for re-infusion that show the best efficacy for the intended purpose^113–115^. Additionally to T cells, other immune cells are utilized, predominantly natural killer (NK) cells^116^ and macrophages^117^. To tackle limitations of autologous products, there are pre-clinical and clinical studies employing allogeneic immune effector cells from healthy donors^118^.

Combinatorial approaches allow for recognizing two or more different epitopes. Logical gating strategies with various degrees of complexity increase specificity and safety, protect healthy cells, and /or avoid therapy resistance^119–121^.

**Progressing manufacturing**: Alternatively to virus-based gene delivery, non-viral strategies and transient CAR expression^122–126^ are examined. Clinical trials with e.g., Sleeping Beauty transposon technology have been conducted or are on their way^127^. Also, strategies for in vivo transduction/transfection are explored^128,129^. Automation, AI-assistance, shorter manufacturing, and point-of-care production can improve the manufacturing process^3^.

### Personalized medicine approaches for engineered adoptive cellular immunotherapies

Current personalized medicine approaches for eACIs lack patient-specific modeling approaches. Available predictive models that support clinicians to assess individual risks and outcomes of CAR T cell patients are population-based models, as they do not continuously update model parameters with each patient’s individual data. For example, the CAR-HEMATOTOX Score enables risk assessment for hematologic toxicity, severe infection, and disease progression following anti-CD19 CAR T cell therapy in refractory/relapsed large B-cell lymphoma (R/R LBCL)^17,18^, and also holds prognostic value for response and toxicity in MM patients treated with BCMA-directed CAR T cells^19^. Additionally, the Endothelial Activation and Stress Index (EASIX) can be applied to R/R LBCL patients receiving anti-CD19 CAR T cell therapy to predict several CAR T-related toxicities^20^. In relapsed/refractory MM, the Myeloma CAR T Relapse (MyCARe) model provides an outcome prediction model for anti-BCMA CAR T cell therapy^16^. As these models are trained and validated for a population and lack periodic updates with individual patient data, they do not qualify as VTs. Nevertheless, as autologous CAR T cell therapies are patient-specific and biologically complex, an elaborate VT model that represents an individual’s (patho)biology, the molecular and cellular characteristics of the retrieved T cells, and the properties of the resulting CAR T cell product would be a favorable solution for improving personalized treatment planning.

## Minimum design specification for virtual twins in engineered adoptive cellular immunotherapies

### Virtual twins for patients eligible for engineered adoptive cellular immunotherapies

**Fig. 1:**
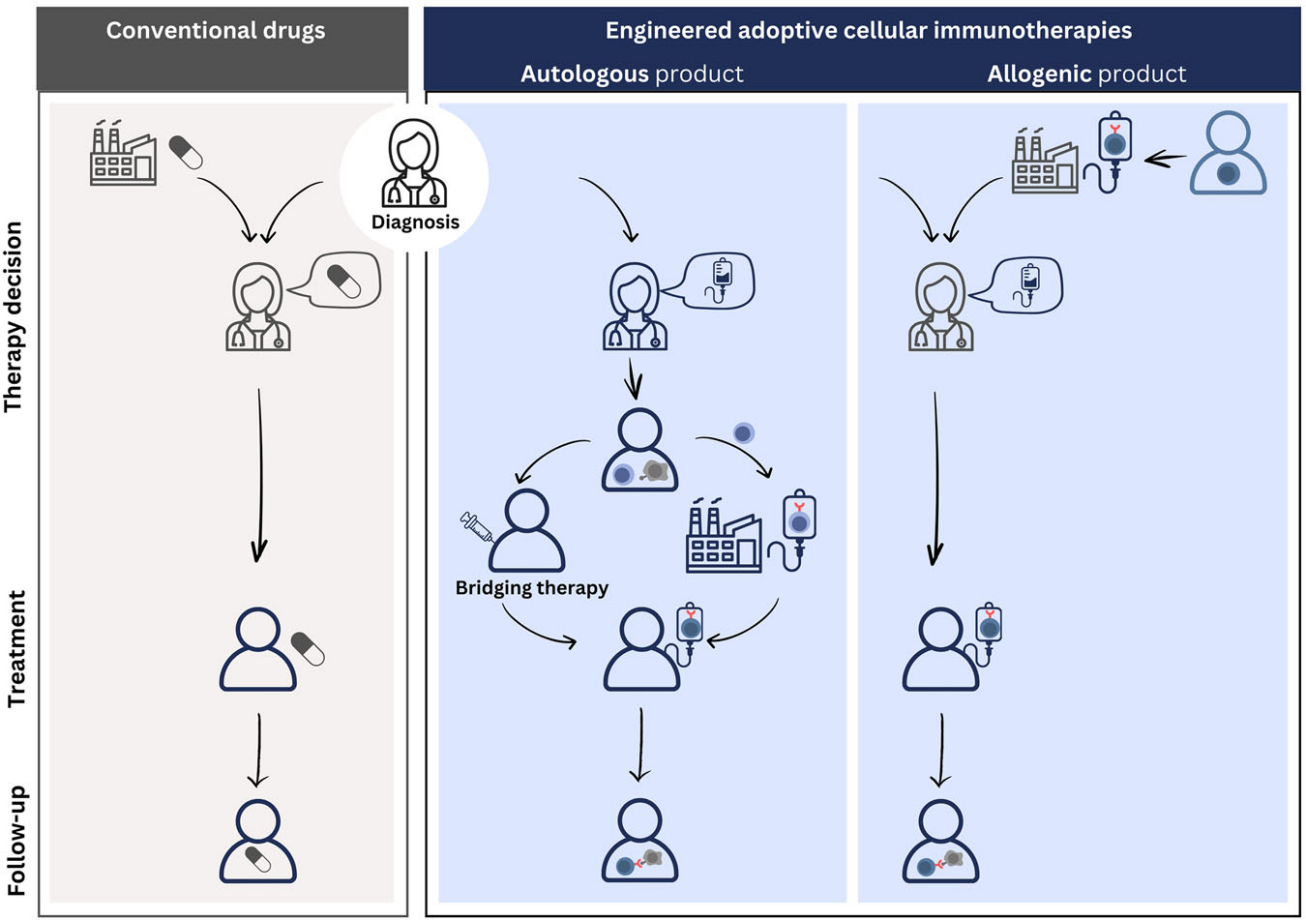
Patient paths for treatment with conventional drugs and engineered adoptive cellular immunotherapies. Upon a newly diagnosed disease or relapse, clinicians select the treatment option that is most likely to benefit the patient at that point in time. In the case of conventional drug treatment (left panel), all patients receive an identical drug that is pre-manufactured and precisely defined. By contrast, autologous engineered adoptive cellular immunotherapies (autologous eACIs) are manufactured individually from the patient’s own immune cells after the patient is found eligible for this option (middle panel). During manufacturing time, the patient receives a bridging therapy. Allogenic eACIs (right panel) are pre-manufactured, off-the-shelf products derived from healthy donors’ immune cells. Still, the products are complex, dynamic, biological systems that may be characterized well, but cannot be defined as precisely as conventional drugs. In both types of eACI treatment, the unique and complex cell product interacts with the patient, leading to dynamic, patient-specific interactions along various biological scales and targeted cells. This warrants that approaches towards virtual twins (VTs) for eACI-eligible patients comprise in silico models of multiple biological scales reflecting (CAR) immune cells before and after manufacturing, as well as models of the immune system and targeted cells throughout therapy. The underlying principle applies to all eACI classes and not only to chimeric antigen receptor (CAR) T cell therapies. The figure was created by the authors using Canva.com.

While the first DTs dedicated to CAR T cell manufacturing processes are under development^3,21^, no VT currently encompasses decision-making tasks across the entire patient journey. Tang et al. propose a five-level roadmap for human body DTs^22^. However, from level three onward, the design specifications only consider perturbations of the human system by conventional drugs, making them too simplistic for eACIs (Fig. 1). With the increasing application of eACIs, their fundamental differences from conventional drugs, and the requirement that VTs should be deliberately designed for specific contexts of use, it is vital to establish the minimally required design specifications for eACI-VTs. Following the definition of VT components^23^, we outline these specifications for eACIs.

### Minimum data categories required in observations of the real-world instance

The efficacy and toxicity of engineered T cells are influenced by factors spanning multiple biological scales^14,15^. To accurately model eACI-introduced changes in patient biology, it is essential to collect not only longitudinal high-level laboratory and clinical values, as is done for conventional drugs, but also longitudinal data on multiscale processes in (CAR) T cells and patient organs.

Therefore, we postulate three minimal required observation categories for an eACI-VT. First, longitudinal multiomics at the single-cell level are needed to measure intra- and intercellular processes influencing, for example, T cell activation, expansion, exhaustion, genotoxicity, on-target/off-tumor binding, immunosuppressive environment, or imbalances in (CAR) T cell clones^24,25^. In this context, the rapid advancement and growing adoption of (single-cell) multiomics technologies are highly promising^26,27^. Without such molecular and cellular data, eACI-VTs cannot infer decisions related to (CAR) T cells and their target cells. Second, longitudinal observations along the organ and body scale, such as CAR T cell expansion and persistence, response to treatment, comorbidities, and side effects, are required. They can be assessed through laboratory data, electronic health records (EHRs), imaging technologies, and sensors. For example, flow cytometry is routinely used to track CAR T cell numbers in peripheral blood, while medical imaging is valuable for monitoring tumor volume during CAR T cell therapy^28^, including extramedullary and minimal residual disease in MM^29^. Third, integration of patient-reported outcomes in combination with socio-economic factors like gender, income, education, and geographic location enhances eACI-VT simulations and validation, prevents bias, and increases predictive accuracy. This results in better-informed decisions and personalized treatment plans that reflect real-world diversity and individual health complexities.

As VTs are patient-specific in silico models aiming to accurately represent the patient under real-world conditions, they benefit from integrating these data categories as real-world data (RWD). For this, international standards for data models (Box 2) and data sharing across healthcare and research, including genomic and single-cell multiomics data, must be followed^30,31^. Importantly, clinical disease development is influenced not only by biological mechanisms, but also by medical decisions (e.g., prior lines of therapy, bridging therapy or follow-up treatment)^32,33^. Integrating longitudinal data that reflects this complexity is crucial. International and national patient registries for eACIs-eligible individuals are valuable sources of patient-specific RWD prior and during therapy. They allow the systematic connection of multiscale information, such as lab test results, patient phenotype, treatment history, clinical decision, treatment efficacy and safety, and (long-term) patient outcomes. Transparent access policies and the ability to interact with one another are a prerequisite for seamless integration of data from registries.

Box 2—Data models relevant for eACI-VTs For observational healthcare data as reported in electronic health records (EHRs), the Observational Medical Outcomes Partnership (OMOP) Common Data Model (CDM) enables large-scale population-based studies and patient-level predictions^130^. While implementations exist for eACI-VT-relevant observational data^131,132^, integration of data types describing observations on organ, tissue, intercellular, and intracellular biological scales derived from an individual is challenging, but essential for credible eACI-VTs. OMOP oncology^132,133^, genomic CDM^134^, the ISO/TS 20428:2024 data standard^135^, complement the OMOP CDM to structured clinical genomic sequence data for describing genomic variants in EHRs. The Global Alliance for Genomics and Health (GA4GH) Phenopacket 2.0, approved as the ISO4454:2022 standard, enables description and exchange of records for individual patients and biosamples through different phenotypic features, including molecular data^136,137^, making it particularly interesting for patient-specific models in eACI-VTs. While these data models provide standards for clinical genomics data^31^, standards for single-cell multiomics data models need to be developed^30,138^. They are essential to reduce noise in data used for updating the computational models of an eACI-VT and thus uncertainty in eACI-VT predictions. Approaching standards for harmonized single-cell atlases^138^ is an essential resource for modeling single-cell biology in eACI-VTs, exemplified by standardizing cell-type annotation through a centralized and community-driven platform of the Human Cell Atlas for cell annotations (https://celltype.info/). The exchange of data records between systems is possible by using Fast Healthcare Interoperability Resources (FHIR) implementation guides, which also account for patient- and sample-individual specificities of genomic data and data descriptions^139^. Integration of genomic data into EHRs allows access for eACI-VTs to data at different biological scales^140^. Software components used in federated learning networks must rely on privacy-preserving federated learning solutions^141^ and on community standards for ensuring model accessibility, reusability, interoperability, and reproducibility^83,142^. The first implementation enabling federated learning for single-cell multiomics data was recently published^70^.

### Minimum design specifications for patient-specific models of the virtual representation

**Fig. 2:**
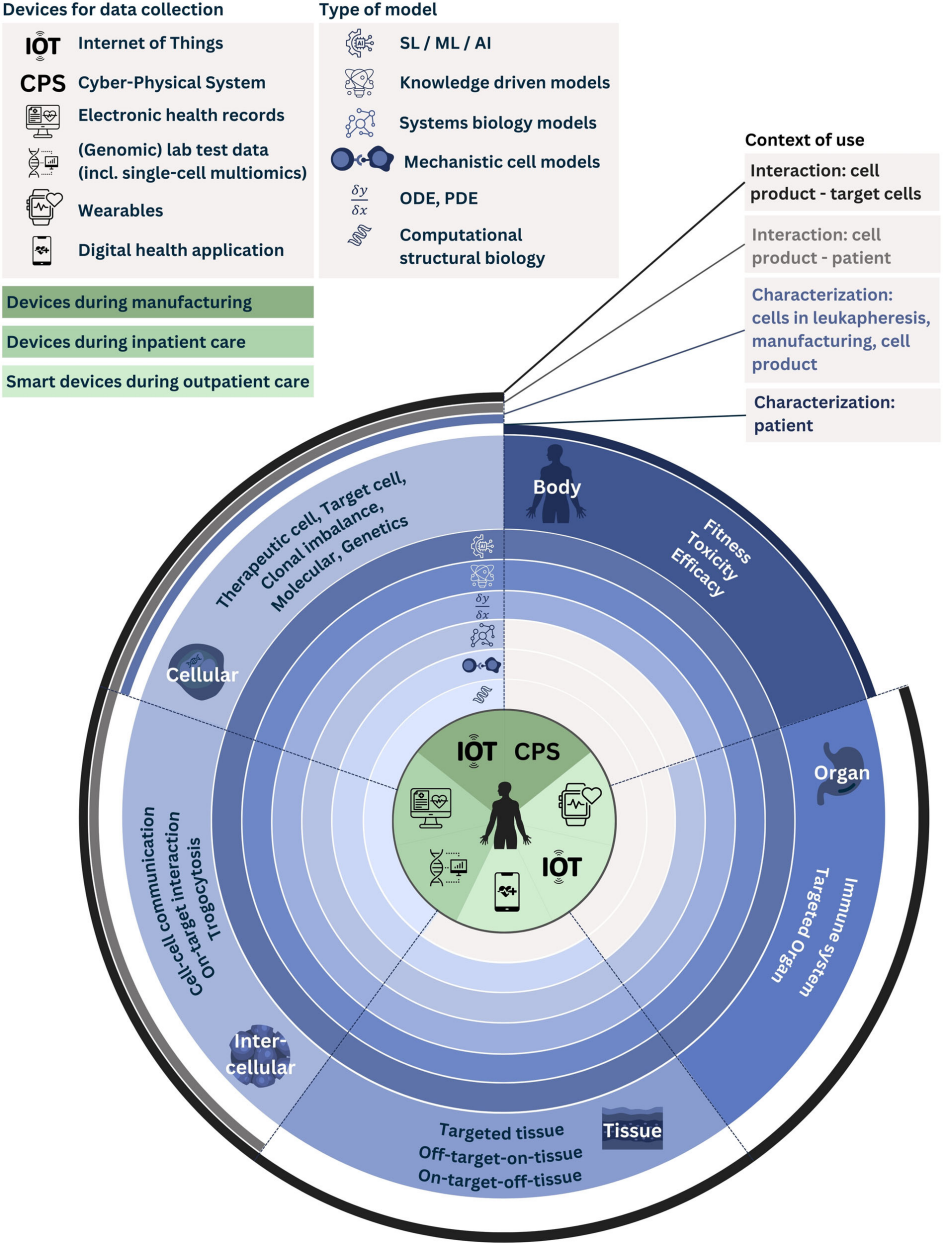
Minimal set of in silico model categories and their context of uses required for the virtual representation of an eACI-VT. CAR T cells are complex, patient-specific (autologous) or donor-specific (allogeneic) therapies. Therefore, it is beneficial to model a patient’s unique (patho)biology alongside the molecular and cellular characteristics of the retrieved T cells and the resulting CAR T cell product. There are four levels of context of uses of an eACI-VT (outer layer): (i) characterizing the patients’/donors’ status prior, the patients’ status during and after therapy, (ii) characterizing the cells during leukapheresis, manufacturing, and in the final cell product, (iii) characterizing the changes of the cell product due the interaction with the patient, and (iv) characterizing the changes of the target cells due to interaction with the CAR T cells. Therefore, the minimal set of in silico models required in an eACI-VT encompass multiple biological scales (middle layer): models for the whole body and for organs reflecting the system-wide status prior to treatment and the impact of treatment, tissue scale, and intercellular scale models for CAR T cell interaction with target cells and the target cells’ tissue during therapy, and cellular scale models that represent intracellular signaling of T cells at time of leukapheresis, (CAR) T cells during manufacturing, the CAR T cells in the medicinal cell product and their target cells. Appropriate in silico model categories that can be used to model events at the different biological scales include (inner layer) system biology models, knowledge-driven models, mechanistic cell models, stochastic models, statistical models (SL), machine learning (ML), and artificial intelligence (AI), ordinary or partial differential equations (ODEs, PDEs), as well as computational structural biology (3D protein structure and 3D/2D RNA structure models). These in silico models receive data generated on different devices and from various systems throughout patient care (central circle). In inpatient care, data from hospital information systems like the electronic health record, as well as lab data and molecular data, fuel the models. Outpatient care provides data via wearables, digital health devices, and the Internet of Things (IoT). During manufacturing, data are supplied via IoT and Cyber-Physical Systems (CPS). The figure was created by the authors using Canva.com.

For a VT to support clinical decisions during eACI, it must provide patient-specific model predictions or simulations for all treatment phases (Fig. 1). This includes initial eligibility, leukapheresis, bridging therapy, CAR T cell manufacturing, infusion, and long-term follow-up. Furthermore, the virtual representation of an eACI-VT must combine in silico models across biological scales relevant to the specific context of use (Fig. 2). Below, we highlight example models suitable for eACI-VTs.

#### Models for intracellular signaling in (CAR) T cells and their target cells

Ma and Gurkan-Cavusoglu^34^ compare different computational methods to model intracellular signaling and provide guidance on selecting the appropriate model for a specific task. Models of intracellular signaling of (engineered) T cells typically rely on existing knowledge of biochemical reaction mechanisms and are often built using continuous and discrete approaches^35–37^. Single-cell multiomics data supports the inference of gene regulation networks^38^. Recently, the advancement of large language models in natural language processing has led to their application in genomics and single-cell studies. The first pre-trained language models for intracellular biology are available. For instance, scBERT^39^ supports cell-type annotation, and scGPT^40^ is the first foundation model covering diverse tasks like cell-type annotation, multi-batch/multiomic integration, perturbation response prediction, and gene network inference. Modeling intracellular biomolecular networks in the context of CAR T cell therapy supports simulation of subcellular processes related to treatment responses by linking a patient’s genotype to their phenotype. A favorable functional status of immune cells is crucial for successful eACI response^41^. Models of this kind could thereby help to use the patient’s individual immune cell status and the status of individual subcellular factors responsible for long-term remission^42^. These simulations could support predictions of manufacturing success^43^ or therapeutic response^25,41^ (Fig. 2). Generative single-cell AI models are also emerging, enabling the creation of patient-specific in silico cells^44^ that may facilitate modeling of immune cell status in in silico clinical trials. Lastly, cellular modeling plays a growing role in DT technologies applications to enhance CAR T cell manufacturing processes^45^.

#### Models for intercellular signaling of CAR T cells and their target cells

Numerous models focus on cell–cell interactions without the details of molecular interactions, investigating the conditions for optimal CAR T treatment responses^36,46,47^. A more recent approach proposes using agent-based models (ABMs) to describe cell–cell interactions within a virtual environment via defined rules^48^. Each agent represents an individual cell, moving and interacting with other cells according to specified rules. Since the interaction of two cells may result in intercellular signaling events, each agent may also contain a mechanistic model, resulting in a hybrid modeling scheme^49^ for which predictions can be made. Integration of patient-specific bulk or single-cell data allows for individual predictions^50^. Furthermore, deep learning models from the AlphaFold family^51^ can predict 3D protein structure. An individual’s genomic sequence data may be used to map genetically observed patient-specific differences to antigens^52^. In turn, these models can be used to predict changes in tumor-associated antigen binding of therapeutics, including the CAR antigen interaction. However, this modeling task remains an area that still needs further improvement in the future^53,54^. Besides protein-protein interaction models, cell–cell communication networks inferred from patient-derived single-cell multiomics^38,55^, spatially resolved, if available, also inform intercellular signaling models.

In CAR T cell therapy, intercellular models simulate the dynamics of the interactions between tumor and CAR T cells^47^ or on-target/off-tumor binding effects^56^, allowing predictions of treatment responses or adverse effects. Deep learning models for protein structure prediction can help identify treatment failures due to individual differences in antigens^52^. In the future, cell-specific DTs combined with a multicellular VT parameterized with data from in vitro or in vivo experiments may support model-informed drug development by simulating treatment success for different CAR T cell designs alongside well-defined experiments. A first variant of this concept systematically explores the multidimensional CAR T cell engineering design space, allowing only the most promising CAR T cell designs to be tested in vitro or in vivo, which reduces the number of experiments conducted and makes the process more cost-effective and ethically favorable^48^.

#### Models for CAR T cell therapy beyond the intra- and intercellular scale

CAR T cell therapy, as a treatment with living cells, affects not only the target cells but also the target’s tissues, organs, and ultimately impacts the whole body^14,15^. For example, cytokine release syndrome, although manageable, is a serious side effect of CAR T cell treatment that leads to systemic inflammation. As such, an ACI-VT must comprise models that go beyond the intra- and intercellular scales. Knowledge-driven modeling methods, such as systems biology maps of immune-related adverse outcome pathways, help assess toxicity profiles^57,58^. As CAR T cells interact with the patients’ immune system, integrating a DT of the human immune system is valuable. Community-driven efforts that build immune system DTs for different human pathologies^59,60^ must therefore be integral to eACI-VTs development. Whole-body models are typically built using multimodal artificial intelligence/machine learning (AI/ML) and are data-driven^22^. Ferle et al. proposed a patient-specific model combining a long short-term memory network with a conditional restricted Boltzmann machine to predict individual blood values over patient trajectories^61^. Maura et al. recently published the first multi-state model for MM that combines genomic and clinical data for individualized prognosis^62^.

Applications of whole-body eACI-VTs include clinical decision-support software that combines guideline-based reasoning with probabilistic assessments of therapy-associated success factors based on real-world evidence^63^. These tools can guide optimal treatment sequences for patients. Additional applications include monitoring during critical and acute medical care shortly after CAR T cell therapy^64^. At later time points, remote monitoring of patients by combining VTs with wearables^65^ could improve outpatient care. First applications detect late cytokine release syndrome^66^ or predict patient-individual blood values^61^. Furthermore, eACI-VTs may also serve as educational platforms for clinicians and nurses, offering realistic and safe environments for learning about CAR T cell eligibility and patient care, as modeled in the digital pathology field^67^.

### Towards credible virtual twins in engineered adoptive cellular immunotherapies

**Fig. 3:**
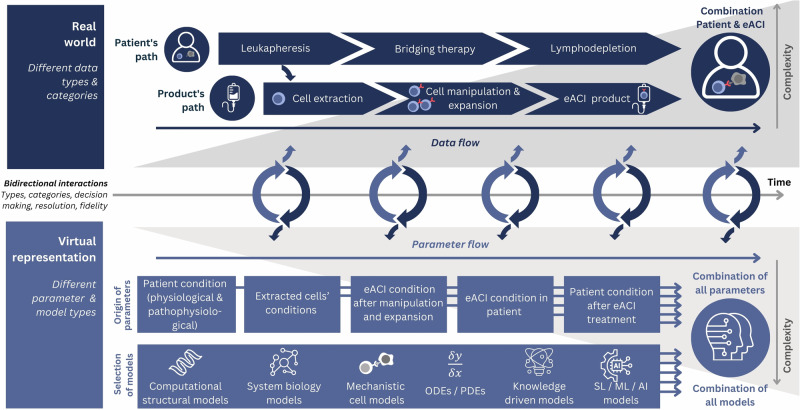
Dynamic and bidirectional interaction between real-world paths through autologous eACI therapy and their virtual representation to generate a virtual twin. A VT differs from population-based models by the bidirectional flow of data between the real-world instance and its virtual representation. Data from the observations of the real-world instance (upper panel) must be collected, processed, and prepared to update the parameters of the virtual representation. This comprises data from the path of the patient, the path of the product, as well as their interaction upon treatment. As different factors on multiple biological scales (patients’ and the cell products’ scales) influence patient trajectory, a virtual representation of interconnected models spanning the whole process of eACI is required (lower panel). This leads to a high need for digitalization and ensuring interoperability. In the virtual representation, parameters dynamically derived from harmonized real-world data covering multiple biological scales are fed into a carefully curated set of models. The updated virtual representation can thus in turn derive decisions with an impact in the real world. AI artificial intelligence, eACI engineered adoptive cellular immunotherapy, ML machine learning, ODEs ordinary differential equations, PDEs partial differential equations, SL statistical learning. The figure was created by the authors using Canva.com.

A VT collects data from observations of its real-world counterpart and processes the data to update the parameters of the virtual representation, which in turn derives decision support for a specific context of use in the real-world (Fig. 3). However, bidirectional interchange is subject to uncertainties in both directions, impacting the credibility, i.e., the trust in “the predictive capability”^68,69^, of a VT. Below, we discuss the main hurdles that likely lead to low eACI-VT credibility and must be addressed during eACI-VT implementation to quantify and control uncertainty arising from model design choices, imprecise parameter fitting, missing information, or biological variance.

First, VT parameters derived from a patient population must build on a representative number of high-quality data entries stemming from the real-world instances. Challenges for eACI-VTs lie in the different data types required to parameterize the virtual representation. These range from electronic health records (EHRs) for patient-level data, patient-reported outcomes, and clinical lab test data—including clinical genomic data—to data derived from single-cell multiomics. While methods exist for integrating clinical genomic data into EHRs, thereby enabling eACI-VTs to seamlessly access data from different biological scales, such approaches have yet to be developed for single-cell multiomics data (Box 2). The systematic collection of data can be addressed by utilizing a decentralized, cloud-based federated learning network, allowing to incorporate datasets from multiple sources while maintaining controlled access to patient data. Initial solutions for integrating clinically derived single-cell multiomics data into a federated learning network have been developed^70^. Organizations participating in such cloud-based platforms should adhere to common data models to allow seamless data integration^71^ (Box 2).

Second, trust in model predictions diminishes when the uncertainty in the observations used for model training/parametrization is high. Single-cell multiomics data is pivotal for informing (multi-)cellular in silico models^69,72^, but using (single-cell) multiomics-derived observations poses a fundamental challenge in model training. Multiomics data is multimodal, often sparse, noisy, and expensive to generate, thus only available in a small number of biological replicates, and has a large signal-to-noise ratio^73^. Moreover, tracking changes of the molecular state of the same cell over time or spatially resolved is only now becoming feasible^74,75^. Therefore, datasets are often unpaired in terms of cell organization and time scale. Observations from “similar” cells need to be integrated to obtain a comprehensive view of a class of comparable cells. This uncertainty in defining a cell’s true ground state presents a significant challenge for data derived in silico models for individual cells or cell–cell interactions. This can be addressed by tailored solutions accounting for the uncertainty in single-cell clustering approaches^76^, expression quantification^77,78^, or cell-type annotation^79^. The problem of missing data can be managed through imputation methods using transfer learning with external reference data^73,79^, underpinning the need for external single-cell reference atlases of engineered immune cells. Additionally, variance in the dimension of the patients’ socio-cultural, economic, and ethical background, as well as sex and gender, influence data quality on all biological scales and should be documented in bioinformatic analyses.

Third, for cell models that are built on general biochemical rules, the causality of input-output relationships is at least approximately understood. In such cases, a mechanistic cell model can be considered trustworthy if intermediate steps and outputs can be inferred from a given input with a reasonably small error margin, e.g., not greater than negligible biological noise or measurement error. Parameter calibration and validation for mechanistic models can be supported by patient-specific in vitro models like patient-derived xenograft (PDX) models, allowing high-throughput analyses^27,80,81^. In cases where patient-specific data is sparse, parameter ranges could be defined and iteratively refined by repeated observations of the readout parameters of PDX models under different conditions. However, the impact on uncertainty quantification of such a nested approach is unknown and must be addressed.

Fourth, given that different factors on multiple biological scales influence the trajectory of patients eligible for CAR T cell therapy, a single model DT is insufficient. Instead, interconnected models are needed to span the whole process in eACIs (Fig. 3), including integration with DTs for the manufacturing process^3,21^. Interactions between models must be thoroughly investigated and validated. Consequently, interoperability across all four levels—technical, syntactic, semantic, and organizational—is essential^82^ but is often not seamlessly addressed by model developers^83^.

Fifth, biomarkers at multiple biological scales represented in the eACI-VT are an essential component for driving meaningful decision-making. In the case of new and innovative therapies like eACIs, validated biomarkers predicting patient outcome remain scarce. Also, due to the complex interaction between drug and host determining outcome on a single patient level, likely composite biomarker signatures allow a more trustworthy prediction^84^. Sensibly, biomarker development can be implemented and advanced in the process of building a VT. Structurally gathering multimodal data across various biological scales from many patients to train and validate the VT enables simultaneously identifying and confirming biomarker signatures from available multiomics data. These biomarkers can then, in turn, be applied in the VT to drive transparent decision-making.

Lastly, AI/ML models need to consider the temporal development of diseases over months and years, which is in contrast to the millisecond-scale of intercellular signaling. Integrating data-driven AI/ML models operating on clinical data with mechanistic models of intracellular signaling and cell–cell interactions remains an open challenge. Potentially, it is possible to extract features of ABM-based simulations, which could be employed as part of the data to train AI/ML models. Solutions to this challenge place high demands on hardware, data storage, and IT infrastructure for emerging digital healthcare technologies (Box 3).

The development of software as medical devices is a detailed process that, if stringently followed, can address some of the above-mentioned uncertainties^85^. Following the life cycle for medical software (IEC 62304), developers can begin with a comprehensive analysis of the technical, medical, ethical, legal, and societal requirements, which can then be refined throughout the development cycles. An incremental, iterative development process allows for the early release of a first prototype that can be tested in selected, relevant environments, enabling co-creation with stakeholders. This approach sets the basis for successfully translating an eACI-VT into a practical and impactful tool for use in both clinical and nonclinical settings. However, challenges that emerge in a hospital setting and with a larger patient population compared to academia, where the eACI-VT was developed, must be addressed during the co-creational process. This includes adhering to legal requirements and ethical considerations with regard to processing sensitive personal data and using AI (Boxes 4 and 5).

**Fig. 4:**
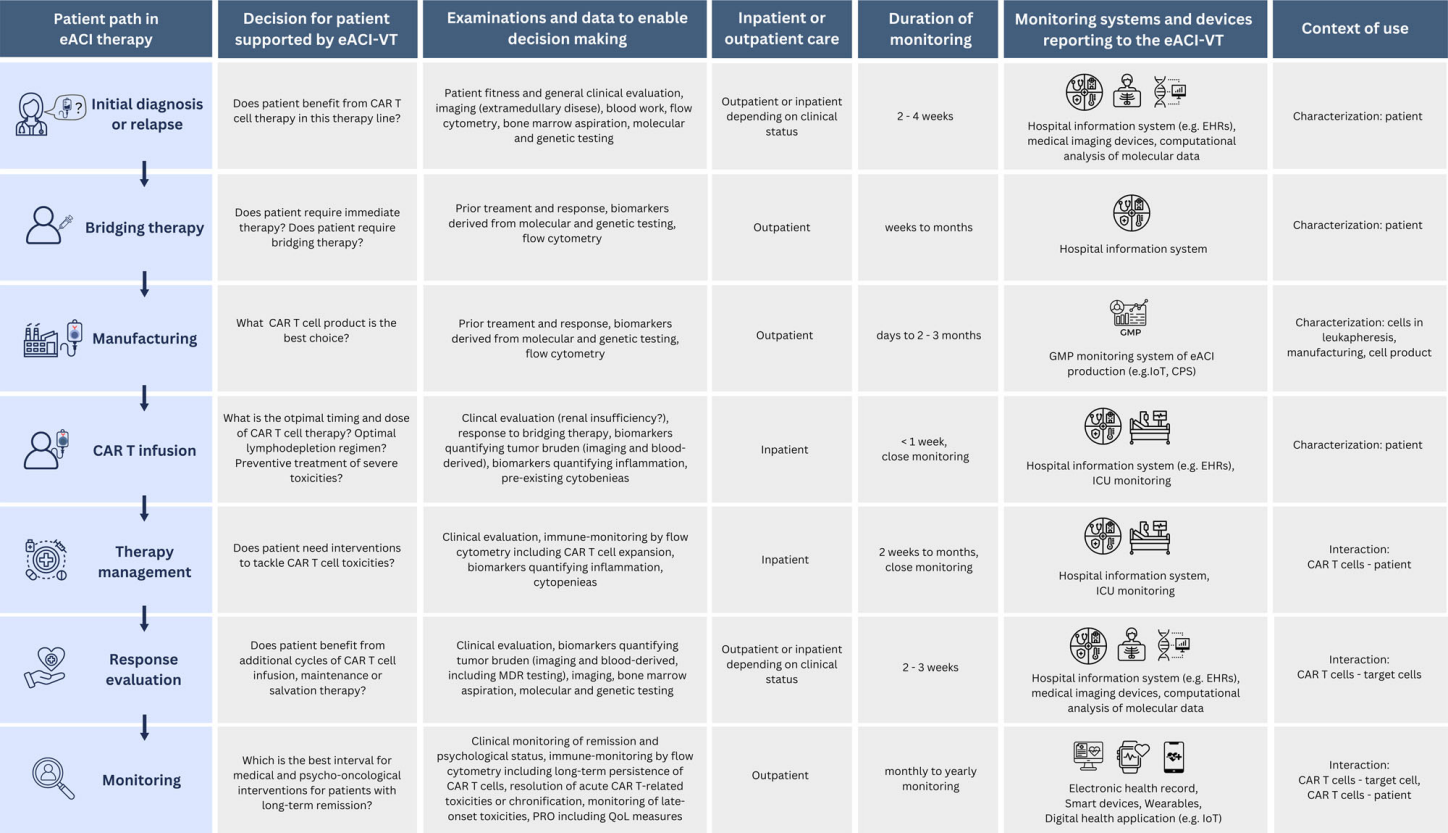
An eACI-VT supports decision-making throughout the path of a multiple myeloma patient eligible for CAR T cell therapy. Throughout the journey of a multiple myeloma patient who is eligible for treatment with CAR T cells, an eACI-VT can support the decisions regarding optimal patient care for each step along the journey. This is facilitated by the frequent update of the eACI-VT with the relevant data on the patient, the product, and their interaction. These data are collected with various devices and systems in the monitoring periods of the patient journey. CAR chimeric antigen receptor, CPS Cyber-Physical System, eACI engineered adoptive cellular immunotherapy, eACI-VT virtual twin for engineered adoptive cellular immunotherapy, EHR electronic health record, GMP good manufacturing practice, ICU intensive care unit, MRD minimal residual disease, IoT internet of things, PRO patient-reported outcome, QoL quality of life. The figure was created by the authors using Canva.com.

Box 3—Technical requirements for eACI-VTs A challenge of integrating data into the eACI-VT is that different decision tasks require updates to the virtual representation at different frequencies. While more extended update frequencies are appropriate for data flow concerning most eACI-VT decisions, some decisions demand more frequent updates. E.g., the occurrence of cytokine release syndrome, a side effect often observed for eACIs, requires instant integration of vital signs. Similarly, decisions during CAR T cell manufacturing require real-time updates of model parameters. Therefore, an eACI-VT must support a technical infrastructure to update model parameters at varying frequencies, ranging from seconds to days and weeks, to ensure efficient model building as well as verification, validation, and uncertainty quantification. A dedicated fine-tuned technical infrastructure builds the basis for integrating model parameters at different biological scales during eACI treatment paths (Figs. 2 and 4). Evolving technologies like smart manufacturing hospitals^143^, Internet of Things (IoT) for healthcare applications^144^, and cyber-physical systems (CPS)^145^ may define the technical infrastructure for eACI-VT software components during eACI manufacturing. Inpatient care for patients treated with eACIs takes advantage of an infrastructure relying on digital health applications in EHRs and devices that capture (genomic) lab test data, including single-cell multiomics. Outpatient care after eACI treatment may build on smart devices like wearables and medical mobile health apps that can be connected with the EHR via IoT solutions. For example, there are first wearable sensors that detect cytokine release syndrome, remotely^146^.

Designing a VT that accounts for minute details ultimately leads to higher fidelity when simulating the real-world scenario^23^. Nevertheless, this simultaneously increases requirements on data storage and computational power to update, verify, and validate the model as well as to estimate uncertainty. Therefore, international computational infrastructures, like the one defined by the European Virtual Human Twin Initiative^10^, are paramount.

Box 4—Regulations crucial for eACI-VTs EACI-VTs operate within an international data protection framework (e.g., European Convention on Human Rights, Convention for the Protection of Individuals regarding the automatic processing of personal data, EU Charter of Fundamental Rights). The General Data Protection Regulation (GDPR) and the European Data Protection Board (EDPB) provide robust structures for safeguarding data privacy and security for utilizing VTs for scientific and medical purposes. Sensitive data, like genetic data, receives increased protection by the GDPR, requiring minimization and security measures, e.g., pseudonymization. Achieving anonymized data as defined by the GDPR is challenging in healthcare, especially for genetic data, as the risk of re-identification is significant^147,148^. For multiomics data integration, a broad patient-explicit informed consent must, in principle, be used^149^. However, the GDPR allows processing of sensitive personal data for scientific research without consent if based on EU or Member State law and if subject to appropriate safeguards. When processing personal data transnationally, compliance challenges arise due to varying national laws and conditions. Effective communication between national legal experts assists stakeholders in developing an appropriate compliance strategy.

In silico models used in virtual representations of VTs must follow recommendations for design, development, and usage of computational models in personalized medicine^150^. Additionally, recent EU instruments such as the AI Act shape the landscape for the application of VTs. While compliance with these regulations is obligatory, adhering to explainable and trustworthy models can also build trust in using VTs for scientific and medical purposes.

AI/ML methods are adaptive, making their use as software/in medical devices (SaMD/SiMD) particularly challenging. The FDA recommends using change management processes that can be reviewed to receive approval on the entire product life cycle^151^. Regulatory sandboxes introduced by the EU monitor approval of AI/ML systems under controlled conditions^152^. Hybrid models should logically adhere to standards for AI/ML-driven and mechanistic models.

Box 5—Ethical and societal implications of eACI-VTs Using VTs to assist clinicians and patients in treatment decision-making offers a chance for developing personalized and highly individual treatment strategies. However, a widespread use of VTs as treatment decision-support tools comes with distinct ethical and societal risks that need to be considered. In part, these risks can be addressed by adhering to ethical and legal requirements for eACI-VTs, e.g., regarding security and protection of the patients’ data or trustworthiness and transparency of dynamic AI-based SaMD/SiMD, as described in Box 4. Other potential ethical and societal implications of regular VT use need to be addressed at different levels. Systems like eACI-VTs are prone to bias, presenting a risk to groups that were under- or unrepresented in the training and validation cohorts. Here, thoughtfully curated and diverse datasets for the various relevant biological and socio-economic features of real-world patients in the setup of the VT are the basis for avoiding bias, improving trustworthy decision-making of the eACI-VT. This can be reached by ensuring co-creation of the eACI-VT with all relevant stakeholders (clinicians, resident doctors, and patients of diverse backgrounds (sex, gender-diversity, etc.)). Furthermore, with the widespread use of VTs in healthcare supporting decision-making, there is a risk of becoming dependent on these AI-based systems rather than seeing them as only a part of a multifactorial process. This could possibly lead to being unable to choose a beneficial therapy without AI, and also raises issues of the liability of treatment choice, putting patients at risk of impaired care. Awareness and training of medical personnel on the chances and risks connected with AI, enabling them to also educate the patients, are crucial for taking VTs as what they are: one tool in a complex toolbox that leads to treatment decision-making in the best interest of the patient.

## How virtual twins improve patient management prior and during treatment with engineered adoptive cellular immunotherapies

EACI-VTs are powerful tools designed to model the complex and unique interactions between administered cells and the patient. They support and educate clinicians and patients throughout the decision-making process, ultimately improving patient outcome and well-being over the entire CAR T cell therapy life cycle (use case of CAR T cell treatment of MM patients: Fig. 4). Of particular importance is the ability of eACI-VTs to integrate patient-specific longitudinal, real-world multimodal data on multiple biological scales. For instance, CD4+ CAR T cells can persist for years, keeping the patient in remission and potentially offering life-long therapeutic benefit^4^. EACI-VTs can anticipate and support future developments in the rapidly evolving field of eACIs, while accounting for time-dependent and dynamic variance in clinical care and diverse patient populations. EACI-VTs enhance knowledge about the mode and mechanism of action underlying these novel treatment options, also supporting the identification of biomarkers, especially those specific to eACI biology^86^, and enhancing post-authorization monitoring. In the development phase, eACI-VTs can simulate treatment response prior to first-in-human studies or aid in clinical trial planning, for example, by sampling synthetic patient populations^87^. Additionally, cases of off-label use and application of out-of-specification CAR T cell products can sensibly be monitored over longer periods of time. This is particularly relevant given recent reports on secondary T cell malignancies following CAR T cell therapy^88,89^. Currently, these cases are investigated by the FDA and the EMA for a possible link between the malignant transformation to insertion site mutagenesis during CAR T cell manufacturing. The FDA recommends life-long monitoring of treated patients^90^. Considering the high costs associated with eACI therapies, which currently limit access, the use of eACI-VTs for accurate patient stratification could have a significant impact on healthcare systems. Applications include cost-effectiveness analysis, which supports performance-based reimbursement models and strategies to reduce long-term costs of eACI treatment, ultimately achieving a more widespread use of this innovative therapy. Overall, VTs^91^, including eACI-VTs, are emerging as a key concept for advancing personalized, risk-adapted decision support in next-generation immunotherapy.

## How virtual twins improve model-informed drug development for engineered adoptive cellular immunotherapies

In addition to supporting clinical decision-making for treating patients, eACI-VTs used in model-informed drug development offer the potential to make eACI development and clinical trials more cost-effective and ethically responsible^92,93^. Clinical trials for regulatory approval of eACIs are typically lengthy^94^. One challenge lies in clinical trial design and recruitment, as access to eACIs is limited, often resulting in underrepresentation of patient diversity in clinical trials compared to post-approval product phases. In silico (clinical) trials could expedite eACIs’ availability by improving predictive accuracy of trials, reducing required sample size, and helping to predict the risk of side effects. These trials rely on synthetic patient populations simulated using computational models parametrized with retrospective RWD. A VT parametrized with RWD can serve as a prior to strengthen the control arm of a clinical trial^93,95^. Patient-specific pharmacokinetic/pharmacodynamic (PK/PD) models reveal dose-exposure-relationships^96^. Ordinary equations can simulate the dynamics of the interactions between tumor and CAR T cells^47^, helping to the reduction of trial sample sizes^92^. Mechanistic models may be used to refine trial designs by avoiding the inclusion of individuals at risk for side effects or by testing efficacy in patient subgroups with characteristics that are not available in the clinical trial population^92,97^. Initial approaches that help to reduce the risk of side effects in first-in-human studies of eACIs combine disease maps, immune-related adverse outcome pathways, and advanced nonclinical in vitro models^58,98,99^. Furthermore, generative AI approaches are expected to play a pivotal role in in silico clinical trials^100^, including models on multiple biological scales. An eACI-VT utilizing generative models can simulate potential clinically relevant outcomes in a study population, hence providing insights about eACI efficacy and safety^101^. Generative AI models for biological cells allow the generation of patient-individual in silico cells^40,44^, which is an essential biological scale for eACI-VTs to model, for example, immune-response upon ACI treatment. Before integrating eACI-VTs into clinical trial workflows, the credibility of each individual in silico model must be rigorously validated. Only after establishing confidence in individual components can the credibility of the overall VT be adequately demonstrated^102^.

## Conclusion and outlook

VTs in healthcare aim to guide biomedical researchers and clinicians in optimizing therapies and treatment regimens tailored to individual patients. Additionally, they empower patients to better understand their unique disease trajectories.

EACIs are “living drugs” comprising individual cells that have complex and dynamic interactions with the host over time, which fundamentally distinguishes them from conventional drugs. Although currently described VT frameworks in healthcare incorporate multimodal data on all relevant biological scales to create single-organ DTs or complex whole-body VTs, they all lack the integration of virtual representations that model the drug and its interaction with target cells that evolve over the course of treatment, spanning decades. Here, we outline the minimum design specifications necessary to adapt VTs for use with “living drugs,” such as CAR T cell therapy. With these minimum specifications, we are pioneering the establishment of a foundational framework for developing eACI-VTs that can accurately simulate the individual pathophysiology of patients eligible for or undergoing eACI. As this complex and potentially revolutionary therapy option expands into earlier lines of therapy and other indications, possibly impacting a broader patient population, we envision a parallel development of eACI-VTs as a tool to support the implementation of true precision health.

## Supplementary information

CERTAINTY Consortium

## Data Availability

No datasets were generated or analyzed during the current study.
