# Supplementary material for: Design specifications for biomedical virtual twins in engineered adoptive cellular immunotherapies
Source: NPJ Digit Med. 2025 Aug 1;8:493. doi: 10.1038/s41746-025-01809-6 (PMC12316993; doi:10.1038/s41746-025-01809-6)
Supplement: Supplementary file 1 — CERTAINTY Consortium [file 41746_2025_1809_MOESM1_ESM.pdf]

CERTAINTY (A CEllulaR ImmunoTherapy VirtuAl Twin for PersonalIzed Cancer TreatmeNT)

Ulrike Weirauch[1], Markus Kreuz[1], Colin Birkenbihl[2], Miriam Alb[3], Maria Quaranta[4], Laurence Calzone[5,6], Sophia Orozco Ruiz[5,6], Stefanie Binder[7], Luise Fischer[8], Solène Clavreul[9], Morine Maguri[9], Maximilian Ferle[1,10], Michael Rade[1], Guillaume Azarias[11], Jay R. Hydren[12], Jakub Jamarik[13], Daniel Schwarz[13], Zsolt Sebastyen[14], Jurgen Kuball[14,15], Georg Popp[1], Chloé Antoine[16], Manon Knockaert[16], Clara T. Schoeder[1,17], David Fandrei[1,8] , Carmen Sanges[3], Vaclovas Radvilas[18], Nico Gagelmann[19,20], Markus Rückert[11,] , Olaf Penack[21], Stephan Fricke[1,22], Andreas Schmidt[23], Carol Ward[24], Carl Steinbeisser[25], Jean-Marc van Gyseghem[16], Anna Niarakis[26,27], Laurent Garderet[28], Michael Hudecek[3,29], Thomas Neumuth[10,17], Uwe Platzbecker[8], Ulrike Köhl[1,7], Regina Demlova[13], Andreas Kremer[4], Stefan Franke[10], Holger Fröhlich[2,30], Maximilian Merz[8,1], Kristin Reiche[1,7,17], Agnes Vosen[31], Alexander Oeser[8], Alexander Scholz[1], André Gemünd[2], Andreas Weber[11], Anne Funck Hansen[1], Annemiek Markslag[15], Arturo Hurtado[12], Carole Batkai[1], Charlotte Vignal[24], Christian Bauer[4], Christina Kuhn[1], Christina Wuest[8], Christoph Kaempf[1], Conny Blumert[1], Daniel Schneider[10], Denisa Denglerová[13], Dennis Löffler[1], Diane Loening-Martens[9], Eduard Lenner[13], Ekaterina Volevah[13], Emma Martinez-Sanchez[14], Emmanuel Barillot[5], Esmée Van Vliet[14], Farid Keramati[14], František Folber[13], Guillermo Puigventos[18], Horst Schwichtenberg[2], Ilka Heinze[1], Iris Bargallo[18], Jacqueline Nakel[23], Jarl Mooyaart[18], Jason Hannon[24], Jenny Ahlstrom[12], Jerina Hoxha[25], Jitka Blažková[13], Joe Tuffnell[18], Johannes Keller[10], Johannes Klier[1], Juan Capdevila[12], Karen Rosier[16], Kate Morgan[9], Katie Joyner[9], Kristin Reiche[1], Lars Bullinger[32], Laurien Baaij[18], Linda Koster[18], Luuk Gras[18], Malin Roth[2], Marco Bressers[18], Markus Elze[24], Melanie Chaboissier[18], Michal Koščík[13], Miroslav Světlák[13], Nicole Modler[7], Nina Becker[1], Nora Grieb[10], Patrick Born[7], Paul Franz[1], Petr Štourač[13], Petra Boudná[13], Regina Ohmer[23], Riika Lempiaeinen[9], Ryan Miller[12], Saran Pankaew[5], Sascha Mühl[31], Sebastian Schwick[2], Sebastian Siegel[11], Shammi More[2], Stefan Schilling[11], Todd Foster[12], Tuula Rintala[18], Uta Schwarz[4], Vincent Noël[5], Zdenka Baresova[13], Zuzana Dostalova[11].

[1] Fraunhofer Institute for Cell Therapy and Immunology IZI, Leipzig, Germany,

[2] Fraunhofer Institute for Algorithms and Scientific Computing SCAI, Sankt Augustin, Germany,

[3] Department of Internal Medicine II, Chair of Cellular Immunotherapy, University Hospital Würzburg, Würzburg, Germany,

[4]Information Technology for Translational Medicine (ITTM) S.A., Esch-sur-Alzette, Luxembourg,

[5] Institut Curie, PSL Research University, INSERM, U 1331, Mines Paris Tech, F-75005, Paris, France,

[6] INSERM, U900, F-75005, Paris, France,

[7]Institute for Clinical Immunology, University Hospital of Leipzig, Leipzig, Germany,

[8]Department of Hematology, Hemostaseology and Cellular Therapy, University Hospital of Leipzig, Leipzig, Germany,

[9] Myeloma Patients Europe aisbl, Brussels, Belgium,

[10] Innovation Center Computer Assisted Surgery, Universität Leipzig, Leipzig, Germany,

[11]TriNetX Oncology GmbH, Freiburg, Germany,

[12]HealthTree Foundation Inc., Lehi, USA,

[13] Masaryk University, Faculty of Medicine, CREATIC, Brno, Czech Republic,

[14]Center for Translational Immunology, University Medical Center Utrecht, Utrecht University, Utrecht, Netherlands,
[15]Cellular Therapy ad Immunobiology Working Party (CTIWP) of the European Society for Blood and Marrow Transplantation (EBMT), Leiden, Netherlands,
[16]Research Center Information, Law and Society, University of Namur, Namur, Belgium,
[17]Center for Scalable Data Analytics and Artificial Intelligence (ScaDS.AI), Dresden/Leipzig, Germany,
[18]European Society for Blood and Marrow Transplantation (EBMT), Leiden, Netherlands,
[19]Department of Stem Cell Transplantation, University Medical Center Hamburg-Eppendorf, Hamburg, Germany
[20]Chronic Malignancy Working Party, Multiple Myeloma Sub-committee chair CAR-T, of the European Society for Blood and Marrow Transplantation (EBMT), Leiden, Netherlands,
[21]Department of Hematology, Oncology and Tumorimmunology, Charité – Universitätsmedizin Berlin, corporate member of Freie Universität Berlin and Humboldt-Universität zu Berlin, Berlin, Germany,
[22]Medicine Campus MEDiC of the Dresden University of Technology at Klinikum Chemnitz gGmbH, Chemnitz, Germany,
[23] Singleron Biotechnologies GmbH, Cologne, Germany,
[24]F. Hoffmann-LaRoche AG, Basel, Switzerland,
[25] Collaborate Project Management, Munich, Germany,
[26]Université Paris-Saclay, Laboratoire Européen de Recherche pour la Polyarthrite rhumatoïde - Genhotel, Univ Evry, Evry, France
[27]Lifeware Group, Inria, Saclay-île de France, Palaiseau, France.
[28]Hematology and cellular therapy department, Sorbonne Université, Hopital Pitié Salpêtière APHP, Paris, France
[29] Fraunhofer Institute for Cell Therapy and Immunology IZI, Cellular Immunotherapy Branch Site Würzburg, Würzburg, Germany,
[30] Bonn-Aachen International Center for IT (b-it), University of Bonn, Bonn, Germany.
[31]Center for International Management and Knowledge Economy IMW, Fraunhofer, Leipzig, Germany
[32]Charite - Universitaetsmedizin Berlin, Berlin, Germany
